# Increased Sympathetic Cardiac Autonomic Modulation after Two Consecutive Tilt Tests in Women with Polycystic Ovary Syndrome

**DOI:** 10.1055/s-0040-1701467

**Published:** 2020-02

**Authors:** Victor Barbosa Ribeiro, Gislaine Satyko Kogure, Rafael Costa Silva, Hugo Celso Dutra Souza, Rui Alberto Ferriani, Rosana Maria dos Reis

**Affiliations:** 1 Department of Gynecology and Obstetrics, Medical School of Ribeirão Preto, Universidade de São Paulo, Ribeirão Preto, SP, Brazil; 2 Insituto Federal de São Paulo, Jacareí, SP, Brazil; 3 Department of Biomechanics, Medicine and Locomotive Apparatus Rehabilitation, Faculdade de Medicina de Ribeirão Preto, Universidade de São Paulo, Ribeirão Preto, SP, Brazil

**Keywords:** polycystic ovary syndrome, cardiac autonomic modulation, hyperandrogenism, spectral analysis, symbolic analysis, síndrome dos ovários policísticos, modulação autonômica cardíaca, hiperandrogenismo, análise espectral, análise simbólica

## Abstract

**Objective** The present study aimed to analyze cardiac autonomic modulation via spectral and symbolic analysis of heart rate variability (HRV) in women with polycystic ovary syndrome (PCOS) who were subjected to two consecutive tilt tests.

**Methods** A total of 64 women were selected and divided into 2 groups: control (without PCOS), and PCOS. Concentrations of follicle-stimulating hormone, luteinizing hormone, prolactin, estradiol, homocysteine, sex hormone-binding globulin, thyroid stimulating hormone, fasting insulin, testosterone, androstenedione, and 17-hydroxyprogesterone levels, triglycerides, free androgen index (FAI), and homeostasis assessment model (HOMA-IR) were assessed. Cardiac autonomic modulation was evaluated by spectral and symbolic analyses during two consecutive tilt tests (two moments) and supine moments before, between and after (three moments) the tilt tests.

**Results** Women with PCOS had higher fasting insulin, HOMA-IR indexes, testosterone and FAI. Additionally, we observed that the PCOS group had greater sympathetic autonomic cardiac modulation in supine 2, tilt 1, and supine 3 moments compared with controls.

**Conclusion** Women with PCOS had higher autonomic sympathetic cardiac modulation even after a second tilt test. No adaptation to this provocative test was observed. Spectral analysis was more sensitive for identifying differences between groups than the symbolic analysis.

## Resumo

**Objetivo** O presente estudo teve como objetivo analisar a modulação autonômica cardíaca por análise espectral e simbólica da variabilidade da frequência cardíaca (VFC) em mulheres com síndrome dos ovários policísticos (SOP) que foram submetidas a dois testes consecutivos de inclinação.

**Métodos** Um total de 64 mulheres foram selecionadas e divididas em 2 grupos: controle (sem SOP) e SOP. Concentrações de hormônio folículo-estimulante, hormônio luteinizante, prolactina, estradiol, homocisteína, globulina de ligação a hormônios sexuais, hormônio estimulante da tireóide, insulina em jejum, testosterona e androstenediona e níveis de 17-hidroxiprogesterona, triglicerídeos, índice de andrógeno livre (FAI) e homeostase modelo de avaliação (HOMA-IR) foram avaliados. A modulação autonômica cardíaca foi avaliada por análises espectrais e simbólicas durante dois testes de inclinação consecutivos (dois momentos) e momentos supinos antes, entre e após (três momentos) os testes de inclinação.

**Resultados** Mulheres com SOP apresentaram insulina em jejuM, índices HOMA-IR, testosterona e FAI mais altos. Além disso, observamos que o grupo PCOS apresentou maior modulação cardíaca autonômica simpática nos momentos supino 2, inclinado 1 e supino 3 em comparação aos controles.

**Conclusão** Mulheres com SOP apresentaram modulação cardíaca simpática autonômica mais alta mesmo após um segundo teste de inclinação. Nenhuma adaptação a esse teste provocativo foi observada. A análise espectral foi mais sensível para identificar diferenças entre os grupos do que a análise simbólica.

## Introduction

Polycystic ovary syndrome (PCOS) is a disease of endocrine origin that affects ∼ 5 to 16% of women of childbearing age.^1^ In 2003, the Rotterdam consensus proposed that PCOS could be diagnosed after excluding other causes of menstrual irregularity, the presence of ovary cysts, and hyperandrogenism,^2^ and their healthcare providers must be aware of the possible risks and complications of PCOS including those related to the cardiovascular system, and endocrine, metabolic, and body composition parameters.^2^ ^3^ ^4^ ^5^ ^6^

The cause and effect relationship between increased insulin resistance (IR) and excess testosterone is evident in PCOS. This has been associated with increased visceral fat and metabolic syndrome,^4^ ^5^ ^6^ ^7^ which predisposes to the development of metabolic chronic diseases^2^ ^6^ ^7^ and of cardiovascular disorders that directly impair the physiology of normal heart function and are often accompanied by impairments in cardiac autonomic control.^8^ ^9^ ^10^ It has been demonstrated that there is a cardiovascular autonomic imbalance in women with PCOS, with increased autonomic sympathetic cardiac modulation and reduced parasympathetic modulation, which increases the risk of cardiovascular disease.^3^ ^10^ ^11^

Noninvasive, reproducible, and low-cost tests have been used to evaluate autonomic function, specifically, heart rate variability (HRV),^12^ ^13^ ^14^ ^15^ which can be analyzed in both a linear and in a nonlinear manner. On the other hand, the tilt test, which involves autonomic provocations by means of postural changes from supine to orthostatic positions (standing, 90°; passively or actively), allows a better evaluation of the autonomic modulation.^16^ ^17^

To our knowledge, there are no data available on HRV after a second of two consecutive tilt tests, nor on whether symbolic analyses would express results similar to those of spectral analyses. An adaptation of HRV to the second consecutive test in the PCOS group could represent an auxiliary form of training that controls the modulation of the cardiac autonomic cardiovascular system. Thus, the present study aimed to analyze the behavior of cardiac autonomic modulation via spectral and symbolic analyses of HRV in women with PCOS subjected to two consecutive tilt tests.

## Methods

### Participants and Methods

In the present case-control study, women with PCOS and controls with regular menstrual cycles were included. The study sample comprised 64 women, 32 with PCOS and 32 controls (without PCOS), aged between 18 and 37 years old, with body mass indexes (BMIs) between18 and 39.9 kg/m^2^ who did not engage in regular supervised physical activity. Women with PCOS were selected from outpatient clinics of the Human Reproduction Sector of the Department of Gynecology and Obstetrics, Ribeirão Preto Medical School, University of São Paulo, Ribeirão Preto, state of São Paulo, Brazil, and the healthy control group (CG) participants were recruited from the women who had routine gynecological examinations at Basic Health Clinics. Polycystic ovary syndrome was diagnosed using the criteria established by the Rotterdam consensus and was based on the presence of at least two of the following conditions: oligomenorrhea or amenorrhea; clinical and/or biochemical signs of hyperandrogenism; polycystic ovaries detected by pelvic ultrasound.^2^ The inclusion criteria for CG were: without PCOS, menstrual cycles occurring at intervals of between 22 and 35 days; duration of menses from 3 to 7 days.^18^ The exclusion criteria for both groups were: other systemic diseases, smoking, pregnancy, use of medications that might interfere with the hypothalamus–pituitary–ovarian axis, and drugs that definitively interfered with the hypothalamic-pituitary-axis or cardiac autonomic modulation.

The present study was conducted in accordance with the ethical standards set forth in the Helsinki Declaration of 1975 and was approved by the Human Research Ethics Committee of the Clinical Hospital of the School of Medicine of Ribeirão Preto, University of São Paulo, Ribeirão Preto, state of São Paulo, Brazil (case HCRP N. 13475/2009).

### Pelvic Ultrasonography

All of the women underwent transvaginal pelvic ultrasonography examinations using a Voluson 730 Expert machine (GE Medical Systems, Zipf, Austria) to evaluate for polycystic ovaries.^19^

### Biochemical Measurements

The concentrations of follicle-stimulating hormone (FSH), luteinizing hormone (LH), prolactin, estradiol, homocysteine, sex hormone-binding globulin (SHBG), thyroid stimulating hormone (TSH), and fasting insulin were determined using a chemiluminescence assay (IMMULITE 2000 Immunoassay System; Siemens, Munich, Germany). Testosterone, androstenedione, and 17-hydroxyprogesterone (17- OHP) levels were measured using radioimmunoassay (IMMULITE 1000; Siemens, Munich, Germany), and glucose levels were assessed using the glucose oxidase method. Total cholesterol (TC), high-density lipoprotein cholesterol (HDL), and triglycerides (TG) were assessed using an enzymatic method, and low-density lipoprotein cholesterol (LDL) was calculated using the Friedewald formula: [LDL = TC - (HDL + TG/5)].^20^ The free androgen index (FAI) was determined using total testosterone [(nmol/L^−1^)/ SHBG (nmol/L^−1^) × 100],^21^ and IR (insulin resistance) was quantified using the homeostatic model assessment of IR (HOMA-IR) ([fasting glycemia level (mg/dL^−1^) _ 0.05551] x [fasting insulin level (μIU/Ml^−1^)]/22.5).^22^

### Anthropometry and Body Fat

Anthropometric measurements were obtained according to the recommendations of the International Society for the Advancement of Kinanthropometry.^23^ ^24^ ^25^ ^26^ ^27^ ^28^ Height was recorded to the nearest 0.1 cm using a standard anthropometer, and weight to the nearest 0.5 kg using a weight scale (Filizola, São Paulo, SP, Brazil). A nonelastic flexible measuring tape was used to measure waist, hip, and abdominal circumferences, with all measurements taken by a single evaluator and recorded to the nearest 0.1 cm. Waist circumference (WC) was measured at the mid-point between the lower ribs and the iliac crest. Hip circumference was measured around the greatest circumference of the gluteal region, while abdominal circumference was measured just under the umbilicus (umbilical waist circumference). The following anthropometric indices were calculated: BMI (kg/m^2^), calculated by dividing body weight by the square of the height, and the waist-to-hip ratio (WHR), calculated by dividing WC (cm) by hip circumference (cm). The body fat percentage (%) was assessed using a QDR Discovery Series dual energy- X-ray absorptiometry (DXA) device (Hologic, Marlborough; MA, USA) and the 5 Discovery Wi model software (S/N 84826) version 13.0 provided by the manufacturer (Waltham, MA).

### Heart Rate Variability Analysis

Spectral and symbolic analyses of HRV were conducted to evaluate cardiac autonomic modulation, using a custom-made computer software (CardioSeries version 2.4; http://sites.google.com/site/cardioseries). The volunteers were asked not to consume alcohol and caffeine or participate in exercise and to maintain a regular diet over the 48 hours before the examination. Recordings for the spectral and symbolic analysis of HRV were performed using ECG signals (AD Instruments, Sydney, Australia) between 8:00 and 11:00 hours over a 60-minute period according to the following protocol: 20 minutes in the supine position on a special motorized tilt table (orthostatic table), and 10 minutes of recording with initial adaptation of the phase followed by 10 minutes of recording in the supine position. Subsequently, the table motor was switched on and the volunteers were moved passively from the supine position to the orthostatic position (90°) for 10 minutes, returning to the supine position for another 10 minutes. Finally, the volunteers were moved passively a second time, from the supine position to the orthostatic position (90°) for 10 minutes, and returning once more to the supine position, where they remained for another 10 minutes.

For the spectral analysis, the R-R interval (RRi) values obtained were resampled (3 Hz) using cubic spline interpolation to adjust the time interval between heartbeats. These were divided into segments of 512 values each with 50% overlap (Welch protocol). Each RRi stationary segment was subjected to fast Fourier transform, after applying a Hanning window function. The oscillatory components were classified as either low frequency (LF: 0.04–0.15 Hz) or high frequency (HF: 0.15–0.5 Hz). The mean values of the power spectral densities of RRi in both bands (LF and HF) are expressed in absolute units (ms^2^). The relative power (%), also known as normalized units (n.u.) in each frequency band, as well as the LF/HF ratio powers, were calculated by subtracting the very low frequency (VLF < 0.04 Hz) values. The normalization tended to minimize the effect of changes in total power on the LF and HF component values.^12^ ^24^ For this reason, in addition, an individualized analysis of the LF/HF ratio was used to verify the predominance of sympathetic and parasympathetic autonomic modulation in women with metabolic syndrome of both groups, in all the five moments, in order to minimize the effects of confouders.

For the symbolic analysis, the methodology used was described previously,^13^ where the lowest iRR of the highest RRi was subtracted and the resulting delta was divided by six, generating six levels with identical intervals (0 to 5). Subsequently, the distribution of the iRR series was performed according to its duration of time. Each symbolic sequence consisted of the sequential values of three iRRs (a symbolic crack) that were transformed into symbols (0, 1, 2, 3, 4, and 5), according to the level that fit each value of iRR. For the final analysis, the following cracking patterns were used: 1) 0V: unchanged patterns, with three equal symbols, for example (2,2,2) or (5,5,5); 2) 1V: patterns with one variation, that is, patterns with two equal consecutive symbols and the remaining ones were different symbols, for example (3,2,2) and (3,3,2); 3) 2LV: patterns with two variations, with the three symbols forming an ascending or descending ramp, for example, (5,4,2) or (1,2,4); and 4) 2ULV: patterns with two variations in reverse, where the three symbols formed a peak or a trough, such as (3,5,3) or (4,1,2). At the end, the 2LV and 2ULV values were summed and presented as 2V. Previous studies have shown that 0% represents sympathetic cardiac autonomic modulation, 2V% represents parasympathetic modulation, and 1V represents simultaneity of the two modulations.^13^ ^15^

### Statistical Analysis

The Shapiro-Wilk test was used to analyze the distribution of quantitative data, with 95% significance in each of the analyzed variables. For comparison of the anthropometric, biochemical, and HRV variables between groups (intergroup), the *t*-test was used to analyze the parametric data, and the Mann-Whitney U test was used for nonparametric data. For comparisons between the bench press, tilt 1, bench press 2, tilt 2, and bench press 3 (intragroup), a one-way analysis of variance (ANOVA) was used. Data are presented as means ± standard errors of the means (SEMs). The differences were considered significant when *p* < 0.05. SigmaStat 11.0 software (Systat Software Inc., San Jose, CA, USA) was used for the statistical analysis.

## Results

The comparison between PCOS and CG groups with respect to anthropometric data, body fat percentiles, and biochemical parameters are shown in Table 1. The PCOS group had higher values of fasting insulin (*p* = 0.014), testosterone (*p* = 0.036), and HOMA-IR (*p* = 0.012) and FAI (*p* = 0.010) scores comparable to the CG. No differences were observed in the other parameters.

**Table 1:** Comparison of anthropometry, body fat and biochemical parameters among women in the control group (CG) without Polycystic Ovary Syndrome and women with Polycystic Ovary Syndrome (PCOS)

Comparison	CG (*n* = 32)	PCOS (*n* = 32)
Anthropometry parameters		
Age, years	29.4 (0.90)	27.0 (0.93)
Weight, Kg	69 (2.57)	75 (3.04)
Height, m	1.61 (0.01)	1.62 (0.01)
BMI	26.8 (1.02)	28.8 (1.15)
WC, cm	78 (2.00)	89 (2.45)
HC, cm	105 (1.91)	108 (1.94)
WHR	0.74 (0.01)	0.76 (0.01)
Body Fat, %	38.8 (1.23)	40.5 (0.92)
Biochemical parameters		
Total Cholesterol, mg/dL	195 (8.99)	198 (6.36)
Triglycerides, mg/dL	95 (6.86)	128 (12.87)
LDL, mg/dL	123 (7.36)	119 (5.29)
HDL, mg/dL	52 (2.10)	54 (2.04)
Fasting Insulin, mg/dL	5.88 (0.95)	9.34 (1.16)*
Fasting Glycemia, mg/dL	97 (3.22)	100 (3.45)
Homa-IR	1.42 (0.26)	2.36 (0.33)*
Homocysteine, *µmol*/*L*	7.37 (0.25)	7.86 (0.40)
TSH, uIU/mL	2.01 (0.98)	2.48 (1.27)
PRL, ng/ml	13.9 (6.58)	16.1 (16.1)
17-OHP, ng/dL	105 (63)	113 (62)
FSH, uIU/mL	4.26 (0.43)	4.72 (0.50)
LH, uUI/mL	5.58 (1.19)	6.96 (1.21)
Testosterone, ng/dL	66 (4.67)	86 (6.68)*
Androstenedione, ng/dL	102 (5.98)	112 (8.72)
SHBG, nmol/L	65 (6.52)	56 (7.64)
FAI	4.65 (0.54)	7.90 (0.91)*

Abbreviations: %, percentage; *µmol*/*L* / L - micromole / liter*;* 17-OHP - 17-hydroxyprogesterone; BMI, body mass index; CG, control group; cm, centimeters; FAI, free androgen index; FSH, follicle stimulating hormone; HC, hip circumference; HDL, High Density Lipoproteins; HOMA-IR, homeostatic model assessment; LDL, Low Density Lipoproteins; LH, luteinizing hormone; mg/dL, milligrams / decilitre; ng / dL, nanogram / deciliter; ng / ml, nanograms per milliliters; nmol / L, nanomol / Liter; PCOS, polycystic ovary syndrome; PRL, prolactin; SHBG, sex hormone binding globulin; TSH, thyroid stimulating hormone; uIU / mL, international microunits / milliter; WC, waist circumference; WHR, hip waist ratio.

The data are presented in mean and standard deviation.

* *p* < 0.05.

Table 2 shows the comparison between the PCOS and CG groups with respect to the HRV data evaluated at the supine 1, supine 2, and supine 3 moments. No differences were identified at supine moment 1. At supine 2, the PCOS group had higher values for LF (n.u.) (*p* = 0.030) and the LF/HF ratio (*p* = 0.030), and a lower value for HF (n.u.) (*p* = 0.30). This superiority in the PCOS group persisted at supine 3 for all three variables: LF (n.u.) (*p* = 0.020) and LF/HF ratio, (*p* = 0.020) and a lower value for HF (n.u.) (*p* = 0.20). In addition, there was superiority of the PCOS group in the LF value (ms) (*p* = 0.002).

**Table 2:** Comparison of cardiac autonomic modulation through spectral and symbolic analysis in the supine 1, supine 2 and supine 3 periods among control women (CG) without Polycystic Ovary Syndrome and women with Polycystic Ovary Syndrome (PCOS)

	Supine 1		Supine 2		Supine 3	
	CG	PCOS	CG	PCOS	CG	PCOS
Spectral Analysis						
RMSSD, ms	55 (6.3)	54 (5.2)	72 (7.1)	69 (6.7)	70 (6.2)	75(7.5)
iRR, ms	896 (20)	901 (15)	967 (21)	972 (16)	978 (23)	978(17)
LF, ms^2^	728 (145)	883 (113)	910 (152)	1220 (162)	1053 (189)	1582 (193)^B^
HF, ms^2^	1520 (433)	1378 (250)	2385 (570)	2073 (404)	2046 (384)	2367 (478)
LF, n.u.	38 (2.5)	44 (2.7)	35 (2.8)	43 (3.0)^A^	37 (2.4)	47 (3.2)^B^
HF, n.u.	62 (2,5)	56 (2.7)	65 (2.8)	57 (3.0)^A^	63 (2.4)	53 (3.2)^B^
LF/HF ratio	0.70 (0.08)	0.95 (0.12)	0.69 (0.13)	1.02 (0.19)^A^	0.69 (0.09)	1.34 (0.31)^B^
Variance, ms^2^	2942 (633)	3279 (405)	4181 (750)	4428 (626)	4139 (629)	5643 (753)
Symbolic Analysis						
0 V, %	9.7 (1.3)	11 (1.39)	7.8 (1.0)	10 (2.41)	8.7 (1.2)	12 (1.69)
1 V, %	40 (1.2)	44 (0.9)	37 (1.1)	39 (1.1)	38 (1.1)	39 (1.0)
2 LV, %	20 (1.1)	17 (1.0)	20 (1.4)	17 (1.1)	18 (1.2)	17 (1.0)
2 UV, %	36 (2.3)	27 (1.6)	36 (2.2)	33 (1.9)	30 (2.3)	32 (2.0)
2 V Total, %	50 (2.2)	45 (2.0)	54 (2.0)	51 (2.2)	54 (2.0)	49 (2.4)
0V/2V ratio	0.26 (0.06)	0.35 (0.08)	0.18 (0.05)	0.28 (0.07)	0.21 (0.05)	0.34 (0.06)

Abbreviations: %, percentage; CG, control group; HF, High Frequency; LF, Low Frequency; ms, milliseconds; n.u., normalized units; PCOS, polycystic ovary syndrome.

The data are presented in mean and standard error.

Supine 2 - CG vs PCOS (^A^ *p* < 0.05); Supine 3 - CG vs PCOS (^B^ *p* < 0.05).

Table 3 shows the comparisons between the PCOS and CG groups with respect to the HRV data evaluated at tilt 1 and tilt 2 moments. At tilt 1, the PCOS group had higher values of LF (ms) (*p* = 0.025), LF (n.u.) (*p* = 0.009), and LF/HF ratio (*p* = 0.009), and variance (ms) (*p* = 0.026) and lower HF (n.u.) (*p* = 0.009). No differences were observed between groups at tilt 2.

**Table 3:** Comparison of cardiac autonomic modulation through spectral and symbolic analysis in the supine 1, supine 2 and supine 3 periods among control women (CG) without Polycystic Ovary Syndrome and women with Polycystic Ovary Syndrome (PCOS)

	Tilt 1		Tilt 2	
	CG	PCOS	CG	PCOS
Spectral Analysis				
RMSSD, ms	24 (3.0)	21 (1.6)	25 (3.6)	21 (1.7)
iRR, ms	720 (17)	711 (12)	712 (17)	708 (13)
LF, ms^2^	595 (115)	759 (82)^A^	860 (130)	863 (140)
HF, ms^2^	367 (107)	251 (42)	422 (150)	237 (43)
LF, n.u.	66 (2.7)	76 (2.2)^A^	73 (2.5)	78 (2.3)
HF, n.u.	34 (2.7)	24 (2.2)^A^	27 (2.5)	22 (2.3)
LF/HF ratio	2.54 (0.30)	4.88 (0.73)^A^	3.98 (0.49)	5.44 (0.69)
Variance, ms^2^	1579 (246)	1896 (172)^A^	2217 (367)	2206 (231)
Symbolic Analysis				
0 V, %	29 (2.4)	34 (2.10)	33 (2.3)	38 (2.05)
1 V, %	45 (0.9)	46 (0.8)	45 (0.8)	44 (0.6)
2 LV, %	13 (1.0)	10 (0.9)	11 (0.9)	9.2 (0.9)
2 UV, %	9.9 (1.3)	9.6 (0.9)	12 (1.4)	8.6 (0.9)
2 V Total, %	25 (2.2)	20 (1.6)	21 (2.0)	18 (1.6)
0V/2V ratio	2.39 (0.68)	2.74 (0.52)	2.70 (0.50)	3.13 (0.46)

Abbreviations: %, percentage; CG, control group; HF, High Frequency; LF, Low Frequency; ms, milliseconds; n.u. normalized units; PCOS, polycystic ovary syndrome.

Tilt 1 - CG vs PCOS (^A^ *p* < 0.05).

The data are presented in mean and standard error.

Table 4 shows the HRV comparisons (intergroup) between supine 1, tilt 1, supine 2, tilt 2, and supine 3 moments in the CG group. The tilt 1 and tilt 2 moment presented higher values of LF (n.u.), LF/HF, 0V (%), and 0V/2V, and lower values in the square root of the square mean of the differences between adjacent normal RR intervals (RMSSD) and iRR HF (ms), HF (n.u.), variance, 2 LV, 2UV, 2 total V when compared with supine 1, supine 2, and supine 3 moments. When comparing the supine moments 1, 2, and 3 with each other, bench press 3 presented higher values those of than supine 1 in RMSSD, iRR, HF (ms) and variance, and lower values than those of 1V and 2 UV, in addition to a lower value for 2UV in comparison to supine moment 2. Supine moment 2 was superior compared with supine 1 in RMSSD, iRR, HF (ms), variance, and values < 1V. Regarding the comparison between tilt 1 and tilt 2, tilt 2 showed higher values for LF (n.u.), the LF/HF ratio, 0V, 2UV, and the 0V/2V ratio, and lower values for HF (n.u.).

**Table 4:** Cardiac autonomic modulation through spectral and symbolic analysis in the supine 1, tilt 1, supine 2, tilt 2 and supine 3 periods among control women (CG) without Polycystic Ovary Syndrome

	CG (*n* = 32)				
	Supine 1	Tilt 1	Supine 2	Tilt 2	Supine 3
Spectral Analysis					
RMSSD, ms	55 (6.3)	24 (3.0)^J^	72 (7.1)^HI^	25 (3.6)^EG^	70 (6.2)^ACD^
iRR, ms	896 (20)	720 (17)^J^	967 (21)^HI^	712 (17)^EG^	978 (23)^ACD^
LF, ms^2^	728 (145)	595 (115)	910 (152)	860 (130)	1053 (189)
HF, ms^2^	1520 (433)	367 (107)^J^	2385 (570)^HI^	422 (150)^EG^	2046 (384)^ACD^
LF, n.u.	38 (2.5)	66 (2.7)^J^	35 (2.8)^H^	73 (2.5)^EFG^	37 (2.4)^AC^
HF, n.u.	62 (2.5)	34 (2.7)^J^	65 (2.8)^H^	27 (2.5)^EFG^	63 (2.4)^AC^
LF/HF ratio	0.70 (0.08)	2.54 (0.30)^J^	0.69 (0.13)^H^	3.98 (0.49)^EFG^	0.69 (0.09)^AC^
Variance, ms^2^	2942 (633)	1579 (246)^J^	4181 (750)^HI^	2217 (367)^EF^	4139 (629)^ACD^
Symbolic Analysis					
0 V, %	9.7 (1.3)	29 (2.4)^J^	7.8 (1.0)^H^	33 (2.3)^EFG^	8.7 (1.2)^AC^
1 V, %	40 (1.2)	45 (0.9)^J^	37 (1.1)^HI^	45 (0.8)^EG^	38 (1.1)^ACD^
2 LV, %	20 (1.1)	13 (1.0)^J^	20 (1.4)^H^	11 (0.9)^EG^	18 (1.2)^AC^
2 UV, %	36 (2.3)	9.9 (1.3)^J^	36 (2.2)^H^	12 (1.4)^EFG^	30 (2.3)^ABCD^
2 V Total, %	50 (2.2)	25 (2.2)^J^	54 (2.0)^H^	21 (2.0)^EFG^	54 (2.0)^AC^
0V/2V ratio	0.26 (0.06)	2.39 (0.68)^J^	0.18 (0.05)^H^	2.70 (0.50)^EFG^	0.21 (0.05)^AC^

Abbreviations: %, percentage; CG, control group; HF, High Frequency; LF, Low Frequency; ms, milliseconds; n.u., normalized units.

The data are presented in mean and standard error.

Supine 3 vs Tilt 2 (^A^ *p* < 0.05); Supine 3 vs Supine 2 (^B^ *p* < 0.05); Supine 3 vs Tilt 1 (^C^ *p* < 0.05); Supine 3 vs Supine 1 (^D^ *p* < 0.05); Tilt 2 vs Supine 2 (^E^ *p* < 0.05); Tilt 2 vs Tilt 1 (^F^ *p* < 0.05); Tilt 2 vs Supine 1 (^G^ *p* < 0.05); Supine 2 vs Tilt 1 (^H^ *p* < 0.05); Supine 2 vs Supine 1 (^I^ *p* < 0.05); Tilt 1 x Supine 1 (^J^ *p* < 0.05).

Table 5 shows the HRV comparisons between supine 1, tilt 1, supine 2, tilt 2 and supine 3 moments in the PCOS group. The tilt 1 and tilt 2 moments presented higher values for LF (n.u.), LF/HF, 0V, and 0V/2V, and lower values for RMSSD, iRR, HF (ms), HF (n.u.), variance, 2 LV, 2UV, 2 total V when compared with the supine 1, supine 2, and supine 3 moments. Only LF (ms) and 1V tilt 2 presented inferior and superior values, respectively, in relation to supine moment 2. When comparing supine moments 1, 2, and 3 with each other, supine 3 values were higher than those of supine 1 for RMSSD, iRR, LF (ms), variance (ms), 0V, 2UV, and 2V total and lower values than 1V, in addition to higher values for LF (ms) and variance (ms) in relation to supine moment 2. Supine moment 2 presented superiority in relation to supine 1 in the RMSSD, iRR, LF (ms), HF (ms), variance (ms), 2UV and 2V total and values lower than those of 0V and 1V. Regarding the comparison between tilt 1 and tilt 2, tilt 2 showed higher values for variance, 0V, and the 0V/2V ratio, and lower values than those of 2LV, 2UV, and 2UV total.

**Table 5:** Cardiac autonomic modulation through the spectral and symbolic analysis in the supine 1, tilt 1, supine 2, tilt 2 and supine periods among women with Polycystic Ovary Syndrome (PCOS)

	PCOS (*n* = 32)				
	Supine 1	Tilt 1	Supine 2	Tilt 2	Supine 3
Spectral Analysis					
RMSSD, ms	54 (5.2)	21 (1.6)^J^	69 (6.7)^HI^	21 (1.7)^EG^	75 (7.5)^ACD^
iRR, ms	901 (15)	711 (12)^J^	972 (16)^HI^	708 (13)^EG^	978 (17)^ACD^
LF, ms^2^	883 (113)	759 (82)	1220 (162)^HI^	863 (140)^E^	1582 (193)^ABCD^
HF, ms^2^	1378 (250)	251 (42)^J^	2073 (404)^HI^	237 (43)^EG^	2367 (478)^ACD^
LF, n.u.	44 (2.7)	76 (2.2)^J^	43 (3.0)^H^	78 (2.3)^EG^	47 (3.2)^AC^
HF, n.u.	56 (2.7)	24 (2.2)^J^	57 (3.0)^H^	22 (2.3)^EG^	53 (3.2)^AC^
LF/HF ratio	0.95 (0.12)	4.88 (0.73)^J^	1.02 (0.19)^H^	5.44 (0.69)^EG^	1.34 (0.31)^AC^
Variance, ms^2^	3279 (405)	1896 (172)^J^	4428 (626)^HI^	2206 (231)^EFG^	5643 (753)^ABCD^
Symbolic Analysis					
0 V, %	11 (1.39)	34 (2.10)^J^	10 (2.41)^HI^	38 (2.05)^EFG^	12 (1.69)^ACD^
1 V, %	44 (0.9)	46 (0.8)	39 (1.1)^HI^	44 (0.6)^E^	39 (1.0)^ACD^
2 LV, %	17 (1.0)	10 (0.9)^J^	17 (1.1)^H^	9.2 (0.9)^EFG^	17 (1.0)^AC^
2 UV, %	27 (1.6)	9.6 (0.9)^J^	33 (1.9)^HI^	8.6 (0.9)^EFG^	32 (2.0)^ACD^
2 V Total, %	45 (2.0)	20 (1.6)^J^	51 (2.2)^HI^	18 (1.6)^EFG^	49 (2.4)^ACD^
0V/2V ratio	0.35 (0.08)	2.74 (0.52)^J^	0.28 (0.07)^H^	3.13 (0.46)^EFG^	0.34(0.06)^AC^

Abbreviations: %, percentage; HF, High Frequency; LF, Low Frequency; ms, milliseconds; n.u., normalized units; PCOS, polycystic ovary syndrome.

The data are presented in mean and standard error.

Supine 3 vs Tilt 2 (^A^ *p* < 0,05); Supine 3 vs Supine 2 (^B^ *p* < 0.05); Supine 3 vs Tilt 1 (^C^ *p* < 0.05); Supine 3 vs Supine 1 (^D^ *p* < 0.05); Tilt 2 vs Supine 2 (^E^ *p* < 0.05); Tilt 2 vs Tilt 1 (^F^ *p* < 0.05); Tilt 2 vs Supine 1 (^G^ *p* < 0.05); Supine 2 vs Tilt 1 (^H^ *p* < 0.05); Supine 2 vs Supine 1 (^I^ *p* < 0.05); Tilt 1 x Supine 1 (^J^ *p* < 0.05).

Table 6 shows the LF/HF ratio of the spectral analysis comparisons during supine 1, tilt 1, supine 2, tilt 2 and supine 3 moments in the GC and PCOS group in women in both groups with and without metabolic syndrome. There was no difference between the groups.

**Tabela 6:** Comparison of cardiac autonomic modulation by LF/HF ratio of spectral analysis in the supine 1, tilt 1, supine 2, tilt 2, and supine 3 periods between control women (CG) without Polycystic Ovary Syndrome (with and without Metabolic Syndrome) and women with Polycystic Ovary Syndrome (PCOS) (with and without Metabolic Syndrome)

Position	CG		PCOS		Metabolic Syndrome (MS)	
	Without MS *n* = (26) *n* (%)	MS *n* = (6) *n* (%)	Without MS *n* = (24) *n* (%)	MS *n* = (8) *n* (%)	CG *n* = (6) *n* (%)	PCOS *n* = (8) *n* (%)
Supine 1	0.64 (0.07)	0.97 (0.33)	0.86 (0.10)	1.25 (0.38)	0.97 (0.33)	1.25 (0.38)
Tilt 1	2.64 (0.32)	2.10 (0.80)	5.51 (1.89)	3.00 (0.94)	2.10 (0.80)	3.00 (0.94)
Supine 2	0.56 (0.07)	1.27 (0.61)	0.75 (0.09)	1.81 (0.68)	1.27 (0.61)	1.81 (0.68)
Tilt 2	3.80 (0.50)	4.74 (1.53)	5.99 (0.81)	3.75 (1.20)	4.74 (1.53)	3.75 (1.20)
Supine 3	0.59 (0.06)	1.12 (0.61)	0.96 (0.13)	2.49 (1.14)	1.12 (0.61)	2.49 (1.14)

Abbreviations: CG, control group; MS, metabolic syndrome; PCOS, polycystic ovary syndrome.

The data are presented in mean and standard error.

## Discussion

The present study suggests that there is a significant increase in sympathetic autonomic cardiac modulation at different times and in different positions among women with PCOS who underwent two consecutive tilt tests. Our spectral and symbolic analyses of HRV were performed simultaneously in women with PCOS and were compared with those of women with regular menstrual cycles. Similar to other studies, both groups showed increases in sympathetic cardiac modulation after changes from the supine positions.^16^ ^17^ However, we noted that the responses to the position changes were different between groups and moments.

At the time of bench press 1, there was no difference in the HRV indices between the groups; however, in supine 2 and supine 3, the women with PCOS had greater sympathetic autonomic cardiac modulations compared with those of the CG group. We observed that the PCOS group had higher LF (%) and LF/HF ratio, and lower HF (%). At tilt times, it was observed that this difference only occurred in tilt 1, and again, the PCOS group demonstrated elevated sympathetic cardiac autonomic modulation compared with the CG group, also due to the higher LF (%) and LF/HF ratio and lower HF (%). In fact, this phenomenon is noteworthy, since the PCOS group, in addition to maintaining a predominance of sympathetic cardiac autonomic modulation in relation to the CG during tilt 1, maintained this predominance in the two returns to the supine position (supine 2 and supine 3).

This alteration of sympathetic autonomic cardiac modulation in women with PCOS may be associated with higher serum concentrations of testosterone and fasting insulin, as well as high FAI and HOMA-IR values. Although we did not perform a correlation analysis, previous studies have reported that the increase in sympathetic cardiac autonomic modulation in women with PCOS occurs due to endocrine-metabolic changes, especially hyperandrogenism and insulin resistance, which are both prevalent in PCOS.^3^ ^25^ Through different tests of short-term HRV, the heart rate and standing blood pressure response after deep breathing and isometric grip, Kuppusamy et al^26^ found in women with PCOS an inverse relationship between insulin resistance and the LF/HF ratio, and increased sympathetic autonomic cardiac modulation, reduced HRV, and increased biochemical factors, including insulin and testosterone. Other researchers have also found, similar to our results, an increase in sympathetic modulation in women with PCOS; however, they measured HRV after a different test that was related to mental stress.^27^ More recently, in animal experimentation, the simple neonatal exposure to excess androgens predisposed the animals studied to autonomic imbalance, due to an increase in sympathetic tone. Excess androgen was also associated with cardiometabolic disorders.^28^

It is known that hyperandrogenism and insulin resistance are associated with increased obesity and metabolic disorders,^2^ ^6^ ^7^ ^28^ ^29^ and these changes, over time, can predispose patients to the development of cardiovascular diseases.^16^ ^26^ Therefore, metabolic alterations such as obesity, diabetes, and visceral fat increase are directly linked to autonomic imbalances, especially increases in sympathetic cardiac autonomic modulation.^16^ ^26^ ^30^ ^31^ However, in our study, we did not observe differences in body fat distribution or percentile between the PCOS group and the CG. In addition, although metabolic syndrome (MS) is a confounding factor, as it may reduce HRV,^32^ in our study we did not observe differences when assessing the LF/HF ratio in women with and without MS. However, in the present study, the number of women analyzed with MS is low compared with the others. Therefore, we suggest that further studies be conducted to assess HRV in women with PCOS and MS.

The increase in cardiac sympathetic autonomic modulation in women with PCOS was found in several studies,^3^ ^16^ ^26^ ^31^ which encouraged us to evaluate the response of these women to a second consecutive tilt test, and to conduct two methods of analysis, one linear and one nonlinear. After our analysis and comparison between the groups, only the spectral analysis showed differences between them, with a greater sensitivity of the spectral analysis compared with the symbolic analysis. However, when the intragroup evaluation was performed at different moments and with changes in posture, it was observed that for both the CG and PCOS groups, 0V and the 0V/2V ratio increased when the volunteers leaned to maintain standing position, similar to LF (n.u.) and the LF/HF ratio, conferring to these variables the postulation of sympathetic cardiac autonomic modulation measurements. On the other hand, it occurred in an opposite way with the HF (n.u.), 2UV, 2LV, and 2V total, that is, they decreased as the volunteers tilted and maintained the standing posture. This finding is consistent with the literature, since at rest there is a predominance of parasympathetic autonomic cardiac modulation, with a reverse of this predominance with changes to standing postures.^16^ ^17^

In the intra-group analyses, we observed that cardiac sympathetic autonomic modulation was higher in tilt 2 compared with tilt 1 in both groups, after detection of LF (%), LF/HF ratio and HF reduction (%) in the CG group and 0V increase and 0V/2V ratio and reduction of 2UV and 2V total in the PCOS and CG groups. However, as reported, on returning to supine position 3, the PCOS group maintained a sympathetic predominance compared with the CG. As previously mentioned, hyperandrogenism and insulin resistance may have contributed to this response in the PCOS group.^16^ ^25^ ^26^ ^31^ Dutra et al^33^ showed that there are important differences between the autonomic cardiac modulation between men and women, with lower sympathetic modulation (lower LF) and higher parasympathetic modulation (higher HF) in women compared with men. In view of this, we suggest that women with PCOS exhibit a cardiac autonomic modulation response to the tilt test that is more similar to that of men, with greater sympathetic heart modulation and lower parasympathetic modulation compared with the CG women. This “masculinization” of the cardiovascular system in women with PCOS has also been suggested by other authors.^33^ ^34^

In our study, one of the objectives was to identify whether the response to a second consecutive tilt test would promote modifications or adaptations in the cardiovascular autonomic responses that might allow the PCOS group to show values similar to those found in the CG. The literature shows that among people with autonomic alterations, especially those with symptoms of vasovagal syncope, one of the forms of treatment has been repeated tilt training, which could, in part, facilitate improved control of autonomic cardiac modulation. In our study, despite repeating the test, we observed that the increase in sympathetic modulation in the PCOS group persisted compared with the control group on the bench press 3. Thus, the present study showed that repeating the tilt test is not sufficient to alter the autonomic control among women with PCOS similar to that observed in women without PCOS.

The present study was innovative in evaluating the effect of two tilt tests in women with PCOS. However, it was limited because it was not randomized. Thus, we suggest that future studies carry out the randomization process as well as investigate and compare the effect of two tilt tests on other populations.

## Conclusion

We conclude that women with PCOS had higher autonomic sympathetic cardiac modulation even after a second tilt test, and no adaptation to this provocative test was observed. In addition, we observed that the linear spectral analysis method was more sensitive for identifying differences between the groups than the non-linear method of symbolic analysis.
